# Giant cell tumor of the talus: A case report and literature review

**DOI:** 10.1016/j.ijscr.2024.110695

**Published:** 2024-11-29

**Authors:** Papa Amadou Ba, René André Macodou Ndiaye, Souleymane Diao, Badara Diop, Alioune Badara Diouf

**Affiliations:** a Hôpital Principal de Dakar, Senegal; b Hôpital Général Idrissa Pouye, Senegal; c Hôpital Régional de Saint Louis, Senegal; d Hôpital Régional de Ziguinchor, Senegal

**Keywords:** Giant cell tumor, Talus, Arthrodesis

## Abstract

**Introduction and importance:**

Giant cell tumor is a benign primary bone tumor of mesenchymal origin that mainly affects the long bones. Involvement of the bones of the foot is rare with an incidence of 1 to 2 %. We report a case of giant cell tumor of the talus in a 36-year-old man.

**Case presentation:**

A 36-year-old tailor with no past medical history presented with pain in the left ankle. The symptoms had been present for approximately three years. A small, non-inflammatory swelling was observed on the anteromedial side of the ankle. The range of motion of the ankle was normal and painless in all planes. X-rays of the ankle revealed a well-defined lytic lesion at the neck of the talus without periosteal reaction. An MRI was performed, revealing a lytic pattern in the inner half of the talus, which expanded and thinned the cortex without obvious signs of breakthrough. The diagnosis was confirmed by a biopsy, which revealed a giant cell tumor of the talus without any signs of malignancy. Surgical treatment by tibiocalcaneal arthrodesis was performed.

**Clinical discussion:**

Giant cell tumor is a benign tumor that usually occurs in long bones in 85 % of cases, with 10 % of cases reported in the axial skeleton. Its location in the bones of the foot is uncommon, with an incidence ranging from 1 to 2 %.

**Conclusion:**

Giant cell tumor is a benign but locally aggressive tumor. Talectomy associated with arthrodesis is a viable therapeutic alternative for extensive destructive lesions.

## Highlights

• This case report presents a rare instance of a giant cell tumor in the talus of a 36-year-old male, emphasizing the tumor's atypical location and its benign yet locally aggressive nature.
• The patient, with a three-year history of ankle pain, was successfully treated with tibiocalcaneal arthrodesis after a diagnosis confirmed by biopsy.
• This case underscores the importance of recognizing giant cell tumors in uncommon sites and considering surgical options for extensive lesions.

## 1 Introduction

Giant cell tumor (GCT) is a benign primary bone tumor of mesenchymal origin, characterized by the presence of multinucleated giant cells [1]. It accounts for 4 to 9.5 % of all primary bone tumors and 18 to 23 % of benign bone tumors [2]. It is locally aggressive and can affect adjacent bones and joints. It primarily affects the long bones (distal femur, proximal tibia, distal radius), with a predilection for the metaphysis, and rarely involves the bones of the foot, with an incidence of 1–2 % [3]. The treatment for such tumors is surgical. It can be conservative by a carcinological resection associated with an arthrodesis or an arthroplasty or radical by leg amputation.

We report the case of a 36-year-old man with a giant cell tumor of the talus, initially treated with traditional medicine. This work has been reported in line with the SCARE criteria [4].

## 2 Observation

A 36-year-old tailor with no past medical history presented with pain in the left ankle. The onset of the pain was gradual and intermittent, worsening during walking. It was alleviated with rest and analgesics. He could walk independently but with a limp. The symptoms had been present for approximately three years. The patient had previously been treated by traditional healers.

**Fig. 1:**
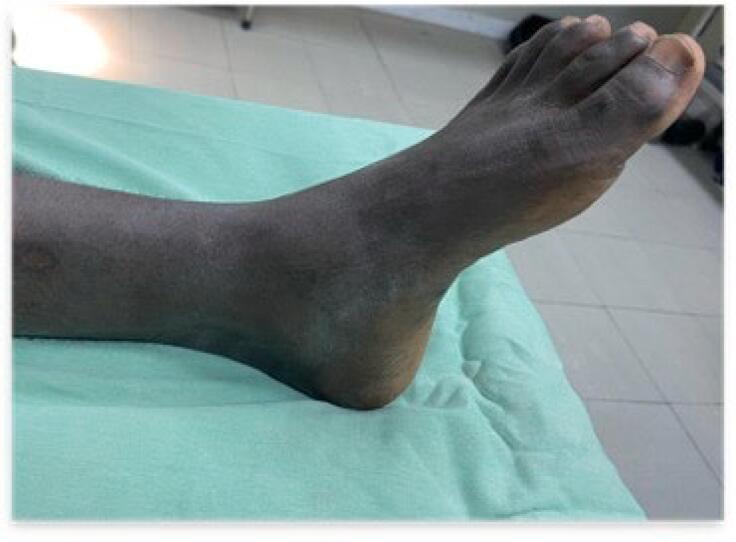
Slight swelling of the anteromedial side of the left ankle.

**Fig. 2:**
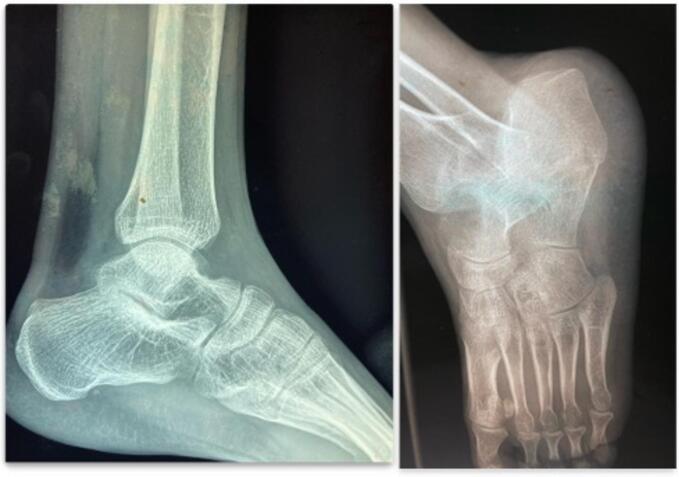
Lateral and 3/4 X-ray of the left ankle.

On clinical examination, the patient was in good general condition. A small, non-inflammatory swelling was observed on the anteromedial side of the ankle (Fig. 1). The range of motion of the ankle was normal and painless in all planes. There was no lymphadenopathy, and the systemic examination of other systems was unremarkable. X-rays of the ankle, both frontal and lateral views, revealed a well-defined lytic lesion at the neck of the talus without periosteal reaction (Fig. 2).

**Fig. 3:**
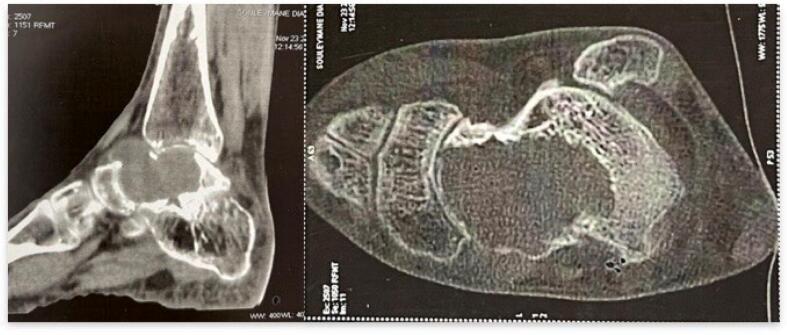
Computed Tomography of the left ankle.

**Fig. 4:**
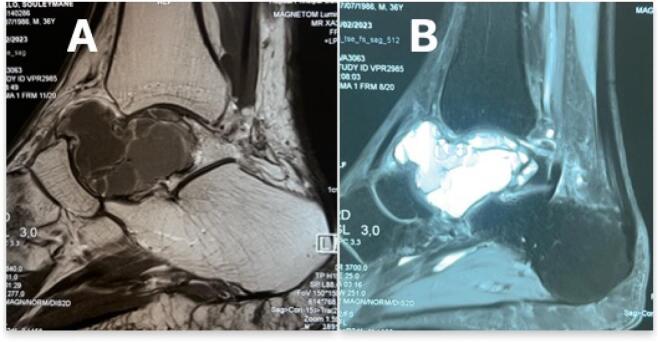
MRI of the left ankle. (A) Sagittal T1 MRI with contrast and fat suppression showing enhancement of the lesion with cortical.

A CT scan confirmed bone lysis involving more than half of the talus (Fig. 3). An MRI was performed, revealing a lytic pattern in the inner half of the talus, which expanded and thinned the cortex without obvious signs of breakthrough. The lesion appeared as a T2 hypersignal, a T1 hyposignal, and was composed of multiple compartments containing fluid levels. The septa were enhanced after contrast injection. This appearance suggested either a giant aneurysmal bone cyst or a giant cell tumor of the talus (Fig. 4).

A thoraco-abdomino-pelvic CT scan, performed to rule out metastasis, was normal. The diagnosis was confirmed by a biopsy, which revealed a giant cell tumor of the talus without any signs of malignancy.

**Fig. 5:**
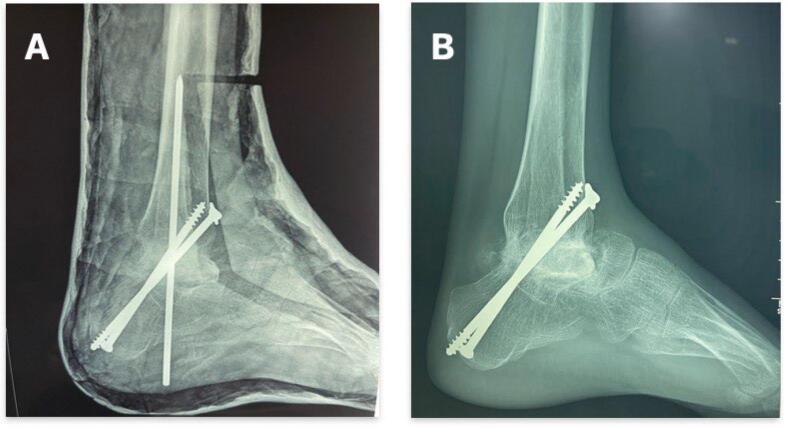
Post-operative X-ray of the left ankle; (A) at 3 months; (B) at 6 months.

**Fig. 6:**
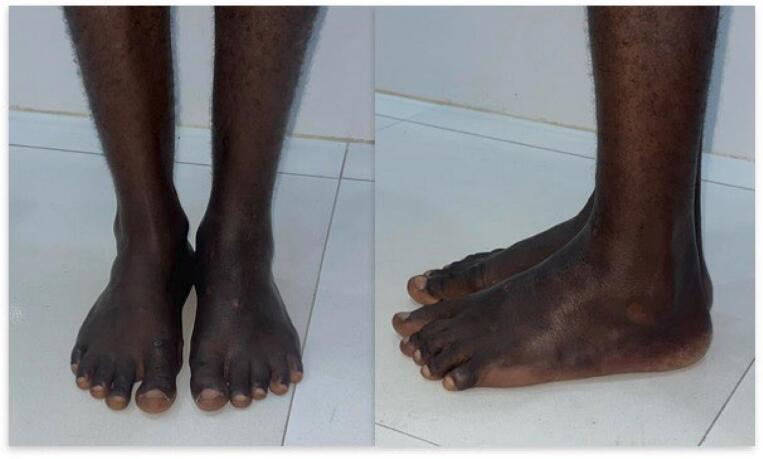
Frontal and lateral X-ray of the ankle 6 months after surgery.

The patient underwent surgery under locoregional spinal anesthesia, which included enucleation of the remaining talus followed by tibio-calcaneal arthrodesis with the interposition of an iliac graft. Fixation was achieved with two divergent tibio-calcaneal cancellous screws, supplemented by a temporary transplantar intramedullary nail. A plaster cast was applied in the operating room. The patient was followed up at 3 weeks, 1 month, and 3 months with standard X-rays (Fig. 5). The cast and intramedullary nail were removed at the 3-month mark after arthrodesis consolidation. Walking was possible with plantigrade support, though with a limp (Fig. 6).

## 3 Discussion

GCT is a benign tumor that usually occurs in long bones in 85 % of cases, with 10 % of cases reported in the axial skeleton [3,5]. Its location in the bones of the foot is uncommon, with an incidence ranging from 1 to 2 % [3]. A literature review on talus involvement revealed very few reported cases, including case reports and case series. However, a retrospective series of 240 cases of GCT reported an incidence of 0.4 % in the talus [6]. GCT of the talus is of particular concern because the lesion is locally destructive. It often recurs even after aggressive surgery and may sometimes undergo malignant transformation [7].

The clinical presentation of GCT typically includes the insidious onset of pain and swelling. This non-specific pain is often misattributed to infection, arthritis, or chronic sprain [8]. GCT frequently occurs in young women aged between 20 and 40 years [9]. To date, the etiology remains unclear.

X-ray is a useful tool for diagnostic orientation, typically showing a radiolucent lytic lesion with bone destruction [9]. The lytic lesion mostly involves the entire talus or its body [[10], [11], [12]]. Some authors have also reported cortical thinning [10,11,13].

Computed tomography (CT) is performed to characterize cortical destruction and the preservation of joint spaces [10,12]. Magnetic resonance imaging (MRI) allows visualization of the extension into soft tissues, ligaments, and capsules. The prognosis for GCT is generally favorable. There is a low risk of malignant transformation [14]. Follow-up chest X-rays are systematically carried out to exclude pulmonary metastases, which are observed in 1–6 % of GCT cases [15]. Overall, GCT is considered a benign bone tumor with a favorable prognosis after appropriate surgical intervention. Current treatment modalities for GCT include curettage and filling, en bloc resection with grafting or arthrodesis, chemical cauterization, talectomy, and, in some cases, amputation.

Arthrodesis is crucial after total talectomy [16]. Amputation is reserved for recurrent forms [10]. Radiotherapy is considered in inoperable cases but is not recommended for completely resected tumors [8]. For giant cell tumors of the talus with well-localized lesions and no cortical rupture, properly performed curettage and bone grafting may be an effective treatment option, preserving near-normal ankle anatomy and function [12]. However, there is a high probability of tumor recurrence after curettage [17].

## 4 Conclusion

GCT is a benign yet locally aggressive tumor, with a high tendency for local recurrence following curettage and bone grafting. Talectomy combined with arthrodesis is a viable alternative treatment for extensive destructive lesions.

## Author contribution

All authors contributed to the conduct of this research work. They read and approved the final version of the manuscript.

## Consent

Written informed consent was obtained from the patient for publication and any accompanying images. A copy of the written consent is available for review by the Editor-in-Chief of this journal on request.

## Ethical approval

Informed consent was obtained from all patients included in the study. Access to the data has been authorized by the hospital's ethics committee, which is responsible for protecting patient information.

## Guarantor

Papa Amadou BA.

## Research registration number

1. Name of the registry: pabaortho.
2. Unique identifying number or registration ID: j4dfkgar.
3. Hyperlink to your specific registration (must be publicly accessible and will be checked): https://www.chictr.org.cn/bin/home.

## Funding

The authors declare that they have not received any funding for the realization of this work.

## Conflict of interest statement

Authors report no conflicts of interest related to this study.
